# Mechanisms of a Patented Chinese Herbal Medicine for Treating Hypothyroidism in *In Vitro* Fertilization-Embryo Transfer: A Combination of Network Pharmacology, Molecular Docking, and Molecular Dynamics Simulation

**DOI:** 10.2174/0113816128364578250212094405

**Published:** 2025-04-15

**Authors:** Chang Liu, Weihuan Hu, Tianyi Zhou, Jue Zhou, Fangfang Wang, Xiaoling Feng, Fan Qu

**Affiliations:** 1 First Clinical Medical College, Heilongjiang University of Chinese Medicine, Harbin, 150040, China;; 2 Department of Traditional Chinese Medicine, Women’s Hospital School of Medicine, Zhejiang University, Hangzhou, 310006, China;; 3 College of Food Science and Biotechnology, Zhejiang Gongshang University, Hangzhou, 310018, China;; 4 Department of Obstetrics, Women’s Hospital School of Medicine Zhejiang University, Hangzhou, 310006, China;; 5 Second Department of Gynecology, First Affiliated Hospital of Heilongjiang University of Chinese Medicine, Harbin, 150040, China

**Keywords:** Chinese herbal medicine, hypothyroidism, *in vitro* fertilization-embryo transfer, network pharmacology, molecular docking, molecular dynamics simulation

## Abstract

**Background:**

Qu’s formula 6 (QUF6), a patented Chinese herbal medicine, is used to treat hypothyroidism in the context of *in vitro* fertilization-embryo transfer (IVF-ET). This research aims to identify the potential bioactive components and elucidate the underlying molecular mechanisms by which QUF6 cures hypothyroidism during IVF-ET.

**Materials and Methods:**

To find the active components of QUF6, the Traditional Chinese Medicine Systems Pharmacology Database and Analysis Platform (TCMSP) and relevant literature were searched. GeneCards and other resources were used to find the targets associated with hypothyroidism and IVF-ET. Using Cytoscape software, the network of interactions was created between the targets and components, the protein-protein interaction (PPI) network was built, and significant targets were verified. Afterward, Gene Ontology (GO) enrichment and Kyoto Encyclopedia of Genes and Genomes (KEGG) pathway analyses were performed on crucial targets. Finally, molecular docking and dynamic modeling were carried out to analyze the essential components and core targets of QUF6.

**Results:**

By creating an interaction network, it was discovered that 92 active components in QUF6 can operate on 25 disease-related targets, with quercetin and other components playing important pharmacodynamic roles. Tumor necrosis factor (TNF), interleukin-6 (IL-6), interleukin-1B (IL-1B), apoptosis regulator Bcl-2 (BCL2), prostaglandin G/H synthase 2 (PTGS2), cellular tumor antigen p53 (TP53), and epidermal growth factor (EGF) were the main targets for the therapy of hypothyroidism. The KEGG pathway enrichment study identified 91 signaling pathways, whereas the GO enrichment analysis identified 1608 entries. Through molecular docking and MD simulations, stable binding was identified between the top five active constituents and the top seven potential targets.

**Conclusion:**

Quercetin, beta-sitosterol, kaempferol, 7-ketocholesterol, and rehmapicrogenin were determined to be the active ingredients in QUF6. The potential mechanism of action for QUF6 may involve modulation of TNF, IL6, IL1B, BCL2, PTSG2, TP53, and EGF to regulate oxidative stress levels, inflammation responses, and apoptosis processes associated with hypothyroidism during IVF-ET.

## 1 INTRODUCTION

Thyroid disease is among the endocrine disorders that affect women frequently. Thyroid hormones play a crucial role in maintaining normal menstrual function and fertility, thereby contributing to successful pregnancy [1]. Hypothyroidism primarily arises from inadequate or absent synthesis, secretion, or biological activity of thyroid hormones, defined by decreasing amounts of free T4 and increased levels of thyroid-stimulating hormone (TSH) [2]. A reduction in the metabolic clearance rates of androstenedione and estrone, along with a decrease in the plasma binding activity of sex hormone-binding globulin (SHBG), are some of the effects of hypothyroidism on female reproductive hormones. As a result, plasma levels of total testosterone and estradiol (E2) decrease [3]. The gonadotropin-releasing hormone (GnRH) response also weakens or delays luteinizing hormone (LH) secretion in women with hypothyroidism. Elevated serum prolactin (PRL) levels may accompany the delayed LH response [4].

Hypothyroidism affects 4% to 5% of people globally. Compared to males, women are more susceptible to hypothyroidism [5]. In the context of pregnancy, the incidence of thyroid pathology increases by six-fold, thus establishing pregnancy as a major associated risk for thyroid disease. Hypothyroidism during pregnancy can cause miscarriage, preterm birth, fetal death, and subfertility if treatment is not received [6]. On the other hand, thyroid hormone levels influence ovulation and oocyte quality *via* interacting with FSH on rat granulosa cells and causing the production of LH/hCG [7]. Conversely, some research revealed that clinical pregnancy is positively correlated with low TSH levels, whereas women with greater TSH have lower rates of fertilization and worse-quality embryos [8, 9]. These studies report that thyroid dysfunction, whether hypothyroidism or hyperthyroidism, can impact fertility and pregnancy outcomes in women.

*In vitro* fertilization-embryo transfer (IVF-ET) is a form of assisted reproductive technology (ART). The preparation for ART involves controlled ovarian overstimulation (COH), which can lead to significantly elevated levels of E_2_. Additional strain is placed on the hypothalamic-pituitary-thyroid (HPO) axis by this rise in E2 levels, thereby compromising thyroid hormone (TH) distribution and dynamics [10]. Numerous studies have reported that the ongoing rise in COH and ART use may have a detrimental effect on thyroid function and pregnancy outcomes [11-13]. In a large cohort study on IVF, researchers divided patients into four groups based on their TSH levels. More unexplained infertility was found in the group with high TSH levels. The cumulative live birth rate and chances of a clinical and continuing pregnancy following IVF were considerably lower for these primary unexplained subfertile women [14]. In addition to impairing fertilization and egg quality, hypothyroidism or thyroid autoimmune disorders also negatively impact placenta and fetal development [15, 16]. Therefore, thyroid dysfunction may have a significant role in determining whether to use standard IVF in women [17].

Traditional Chinese Medicine (TCM) does not have a specific nomenclature for hypothyroidism. Most practitioners advocate that hypothyroidism falls under the categories of “virtual labor,” “virtual damage,” and “sickness.” The underlying cause of hypothyroidism is generally attributed to yang deficiency and emotional internal injury. Its pathogenesis primarily involves yang deficiency in the kidney, spleen, and heart [18]. Kidney yang deficiency is the primary pathogenesis, with spleen yang deficiency and liver qi stagnation as the secondary pathogenesis. In addition, the body is invaded by pathogens, and the healthy qi is damaged, preventing the expulsion of the pathogen. Pathological products, such as endophytic water dampness, phlegm, and static blood, block the body and serve as important pathogenic factors. Qu’s Formula 6 (QUF6) is a patented Chinese herbal medicine owned by the corresponding author’s team that has been developed for the treatment of hypothyroidism in IVF-ET (Chinese National Invention Patent: CN103877409A). It comprises Gardeniae Fructus (Zhizi), *Artemisiae scopariae herba* (Yinchen), Rehmanniae Radix Praeparata (Shudihuang), *Atractylodes macrocephala koidz* (Baizhu), Mori Folium (Sangye), *Lophatherum gracile* (Danzhuye) and *Radix rehmanniae* (Shengdihuang) in specific proportions of 2:4:3:4: 3:2:2 per dose weighing 100 g. In QUF6, the combined effects of various Chinese medicinal herbs work together to warm the kidney, fortify the spleen, drain dampness, and soothe the liver. For oral administration, the medication can be made into granules, powders, decoctions, or capsules. This study used a combination of network pharmacology and molecular docking analysis to examine the therapeutic mechanisms of QUF6 in the treatment of hypothyroidism during IVF-ET.

## 2 MATERIALS AND METHODS

### 2.1 Screening of Active Constituents and Potential Targets of QUF6

The active constituents for Gardeniae Fructus (Zhizi), *Artemisia scopariae herba* (Yinchen), Rehmanniae Radix Praeparata (Shudihuang), *Atractylodes macrocephala koidz* (Baizhu), and Mori Folium (Sangye) of the medication QUF6 were obtained by searching the Traditional Chinese Medicine Systems Pharmacology Database and Analysis Platform (TCMSP; https://old.tcmsp-e.com/index.php) [19]. The screening criteria in TCMSP were set as an oral bioavailability (OB) of ≥30% and a drug-like property (DL) of ≥0.18 [20]. A Bioinformatics Annotation Database for Molecular Mechanism of Traditional Chinese Medicine (BATAMAN-TCM; http://bionet.ncpsb.org/batman-tcm/) [21] was used to identify the medications, revealing the active components and possible targets of *Lophatherum gracile* (Danzhuye) and *Radix rehmanniae* (Shengdihuang). The screening criteria were a *p >* 0.05 and a cutoff value of > 80 [22]. After eliminating duplicate entries from both databases, the protein names were subsequently converted to official gene names based on UniProKBt in the Universal Protein Database (UniProt, http://www.Unitprot.org/) [23].

### 2.2 Identification of Action Targets for IVF-ET and Hypothyroidism

To find possible targets, the terms “hypothyroidism”, “thyroid-stimulating hormone deficiency”, and “*in vitro* fertilization-embryo transfer” were searched as keywords through the GeneCards Database (https://www.genecards.org/), online Mendelian genetic Database (OMIM, https://www.omim.org/), and Drugbank Database (https://www.drugbank.ca/) [24-26]. Following the elimination of duplicate entries for all collected disease targets, a comprehensive list of potential targets associated with the disease was compiled.

### 2.3 Network Visualization

The visualization platform (http://www.bioinformatics.com.cn/) was used to get the intersection targets by entering the possible targets of QUF6, hypothyroidism, and IVF-ET. It is believed that the common genes formed by the intersection of these target genes might be targets for the therapeutic effects of QUF6 in the treatment of hypothyroidismas associated with IVF-ET. A “herb-constituent-target” (H-C-T) network was then created using Cytoscape software (Version 3.7.2, USA) by matching these intersection targets with the active constituents-target data of QUF6 [27].

### 2.4 Protein-protein Interaction (PPI) Network Construction

For protein-protein interaction (PPI) analysis, the intersecting genes previously acquired were entered into the STRING database for further analysis (http://stringdb.org/). Then, the isolated proteins were hidden, the threshold was set as > 0.4, and the TSV file was exported. PPI graphs were drawn using Cytoscape software (Version 3.7.2, USA) [28]. A node’s prominence in the network is indicated by its “Degree” value. The node is more significant if the “Degree” value is higher. After that, the primary active ingredients were sorted by the “Degree” value [29].

### 2.5 Gene Ontology (GO) and Kyoto Encyclopedia of Genes and Genomes Pathway Enrichment (KEGG) Analysis

Gene Ontology (GO) and Kyoto Encyclopedia of Genes and Genomes (KEGG) enrichment analysis were performed on intersection targets using the DAVID database (https://david.ncifcrf.gov/home.jsp), with *p*<0.05 being statistically significant [30]. Bar charts and bubble charts were drawn using online tools from the bioinformatics plotting website after saving all the results (http://www.bioinformatics.com.cn/).

### 2.6 Molecular Docking Analysis

The top 5 key active constituents and the top 7 core targets were selected for molecular docking analysis using AutoDockTools 1.5.7 and PyMOL software (Version 2.1, USA) [31, 32]. The core targets and their 3D structures were obtained in PBD format files from the Protein Data Bank database (PDB, https://www.rcsb.org/) in the PPI network [33]. The TCMSP Database provided the Mol2 files for the top five active components of QUF6. Small molecules were preprocessed using AutoDock by reading in small molecules, adding hydrogens, calculating charges, specifying them as docking small molecules, and outputting them as a pdbqt format file. PyMOL was used to remove solvent and heteroatoms from the protein’s PDB file. Afterward, large molecules were preprocessed using AutoDock by reading in the protein, adding hydrogens, calculating charges, setting rotatable bonds, specifying them as docking proteins, and outputting them as a pdbqt format file. To set up the docking box, the pqbqt files of the proteins and small molecules were entered into AutoDock. The receptor protein was set to semiflexible docking, the genetic algorithm was selected, and the maximum number of evals was set as the medium [34, 35]. The docking event with the lowest binding energy was considered as the final outcome.

### 2.7 Molecular Dynamics (MD) Simulations

MD simulations were conducted using GROMACS 2020.3 software [36]. The parameters and topologies of the proteins and ligands were generated using the amber99sb-ildn force field and the general Amber force field, respectively. The protein atoms were at least 1.2 nm from the water box’s closest border, and then the right quantity of Na^+^ and Cl^-^ was added to balance the simulation system’s charge. Using the steepest descent approach for energy minimization, the solute was limited in the NVT ensemble. The system was gradually heated from 0 K to 300 K and then equilibrated at 300 K and 1 Bar in the NPT ensemble. A 100 ns molecular dynamics simulation was run on the complex, and the simulation trajectory was preserved for further analysis. The complex’s radius of gyration (Rg), solvent-accessible surface area (SASA), hydrogen bonds (H-bonds), root mean square deviation (RMSD), and root mean square fluctuation (RMSF) were computed using the MD simulation data. Rg can be used to describe the compactness of a protein structure and the changes in the looseness of a protein’s peptide chain throughout the simulation [37]. One crucial structural property metric associated with protein structure and function is the solvent-accessible surface. A common metric for characterizing the extent of residue exposure on the protein surface or within proteins is SASA [38]. RMSD is a method to calculate the root mean square deviation of the structure between two specified time points and to analyze the kinetic behavior of a protein by time series [39]. RMSF indicates the degree of freedom of movement of each atom in the molecule [40]. Trajectories were visually shown, analyzed, and animated using PyMOL 2.4.1 and the visual molecular dynamics (VMD) program version 1.9.3 [41]. The compound’s binding free energy was determined using gmx_mmpbsa (http://jerkwin.github.io/gmxtool) [42].

## 3 RESULTS

### 3.1 Screening of Active Constituents and Potential Targets of QUF6

Through retrieval and screening, we identified 91 active constituents and 652 potential targets of QUF6. Among these, Gardeniae Fructus (Zhizi) contained fifteen active constituents, *Artemisiae scopariae herba* (Yinchen) contained thirteen, *Rehmanniae radix Praeparata* (Shudihuang) contained two, *Atractylodes macrocephala koidz* (Baizhu) contained seven, Mori Folium (Sangye) contained twenty-nine, *Lophatherum gracile* (Danzhuye) contained six and *Radix rehmanniae* (Shengdihuang) contained twenty-seven. Detailed information regarding the active constituents of QUF6 is presented in Table **1**.

### 3.2 The Targets of Hypothyroidism and IVF-ET

Specifically, we retrieved 3748, 45, and 69 targets linked to hypothyroidism from the GeneCards, OMIM, and DrugBank databases. Following deduplication, 3830 linked targets were left. The GeneCards and OMIM databases yielded 155 and 571 IVF-ET-linked targets, respectively, while 716 relevant targets were eliminated.

### 3.3 Construction of H-C-T Network

**Fig. (1):**
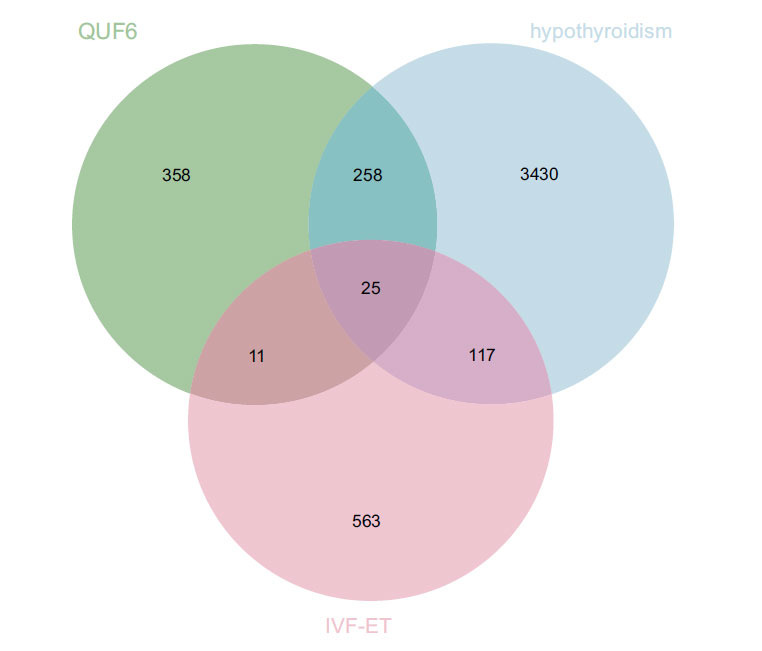
The Venn diagram of the intersection genes of the active constituents of QUF6, the hypothyroidism-related targets, and IVF-ET-related targets.

**Fig. (2):**
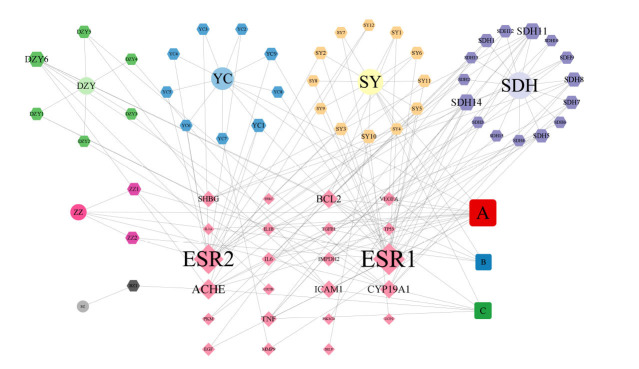
“Herb-constituent-target” (H-C-T) network of QUF6. The drug is named after its Chinese **abbreviations**. Three of the recurring active constituents are designated as A, B and C. ZZ: Gardeniae Fructus (Zhizi); YC: *Artemisia Scopariae Herba* (Yinchen); BZ: *Atractylodes* *macrocephala koidz* (Baizhu); SY: Mori Folium (Sangye); DZY: *Lophatherum Gracile* (Danzhuye); SDH: *Radix Rehmanniae* (Shengdihuang); A: Quercetin; B: Beta-sitosterol; C: Kaempferol.

Twenty-five intersecting genes were found (Fig. **1**) and added to the H-C-T network, which had 77 nodes (6 TCM nodes, 46 compound nodes, and 25 target gene nodes) and 156 edges (Fig. **2**). Greater node relevance is indicated by larger degree values. Table **2** displays the top five active constituents with their corresponding degree values. Table **3** provides more details on the nodes that make up the H-C-T network.

### 3.4 PPI Network

**Fig. (3):**
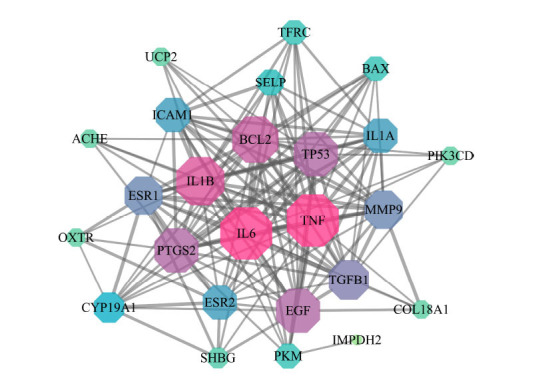
The Protein-protein interaction (PPI) network. The node size is proportional to its “Degree”. The associated “Degree” values increase when the nodes are colored in a radial fashion from green to pink.

The construction of the PPI network was carried out using 25 intersection targets (Fig. **3**), resulting in the acquisition of 25 nodes and 149 edges for subsequent analysis. Higher “Degree” nodes suggest that they could be important to the network. Tumor necrosis factor (TNF), interleukin-6 (IL-6), interleukin-1B (IL-1B), apoptosis regulator Bcl-2 (BCL2), prostaglandin G/H synthase 2 (PTGS2), cellular tumor antigen p53 (TP53), and epidermal growth factor (EGF) were the top seven main targets determined by the “Degree” value.

### 3.5 GO and KEGG Analyses

The GO enrichment analysis encompassed a total of 1717 GO function items, comprising 1608 biological processes (BP), 54 cell components (CC), and 55 molecular functions (MF). Fig. (**4A**) illustrates the top ten significantly enriched terms in the BP, MF, and CC categories. BP was mainly enriched in response to organic substances, oxygen-containing compounds, chemical stimuli, and lipids. CC was mainly enriched in the endomembrane system, extracellular region part, secretory granule, and cell surface. MF was mainly enriched in identical protein binding, cytokine activity, cytokine receptor binding, and growth factor receptor binding.

A total of 91 KEGG pathway enrichment items were identified, primarily involving the signaling pathways related to advanced glycation end products (AGE) and their receptor (RAGE) signalling pathway in diabetic complications, fluid shear stress, and atherosclerosis, lipid and atherosclerosis, pathways in cancer, TNF signaling pathway, hypoxia-inducible factor 1 (HIF-1) signaling pathway, mitogen-activated protein kinase (MAPK) signaling pathway, and interleukin-17 (IL-17) signaling pathway. The top 20 items were selected for further analysis, as shown in Fig. (**4B**).

### 3.6 Molecular Docking Analysis

Using AutoDock software, molecular docking were performed between the key hypothyroidism targets (TNF, IL6, IL1B, BCL2, PTSG2, and TP53) and the key constituents of QUF6 (quercetin, beta-sitosterol, kaempferol, 7-ketocholesterol, and rehmapicrogenin). Favorable docking activity was indicated by a binding energy of less than -5.0 kcal/mol, whereas spontaneous docking between the ligand and receptor was indicated by a binding energy of less than 0 kcal/mol [43].

**Fig. (5):**
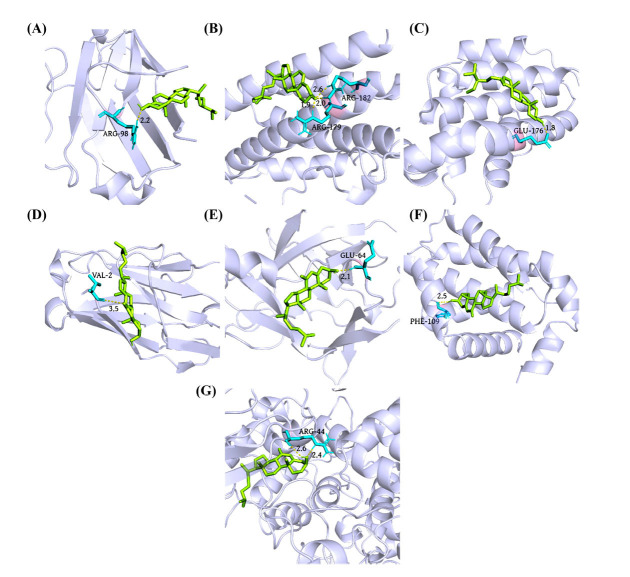
Molecular docking energy of core constituents of QUF6 and key targets. (**A**) TNF-B; (**B**) IL6-B; (**C**) BCL2-B; (**D**) TNF-DZY6; (**E**) IL1B-DZY6; (**F**) BCL2-DZY6; (**G**) PTGS2-DZY6.

As presented in Table **4**, all 34 pairs exhibited satisfactory docking results except for EGF with SDH14. The number of hydrogen bonds, position, and length of each pair are mentioned in Table **5**. Among these, seven docking couples with binding energies below -8 kcal/mol were formed by TNF-B, TNF-DZY6, IL6-B, IL1B-DZY6, BCL2-B, BCL2-DZY6, and PTGS2-DZY6. These pairs were able to generate several structurally stable hydrogen bonds. These seven docking findings are presented using the PyMOL (Fig. **5**). As depicted in Fig. **5C**, beta-sitosterol formed two hydrogen bonds with ARG-182 in BCL2, exhibiting bond lengths of 2.6 and 2.0, respectively. Additionally, it established a single hydrogen bond with ARG-179 (bond length: 1.9). In Fig. **5G**, PTGS2 exhibited the highest affinity towards 7-ketocholesterol, which engaged in two hydrogen bonds with ARG-44 (bond lengths: 2.6 and 2.4).

### 3.7 The Molecular Dynamics (MD) Simulations

RMSD signifies the stabilization of corresponding atoms, whereas fluctuating RMSD indicates variability. After 20 ns, the protein-ligand complex reached equilibrium, as shown in Fig. (**6A**). Protein-ligand recombination SASA values did not vary substantially throughout all recombination simulations, as illustrated in Fig. (**6B**), suggesting consistent protein-ligand binding. As shown in Fig. (**6C**), stable protein-ligand binding demonstrated that the protein Rg value did not significantly alter during the complex MD simulation process when SASA findings were inhibited. As demonstrated in Fig. (**6D**), the average hydrogen bond numbers of BCL2-B, BCL2-DZY6, IL-1B-DZY6, IL-6-B, PTGS2-DZY6, TNF-B, and TNF-DZY6 were 0.34, 0.63, 0.42, 0.04, 0.50, 0.04, and 0.07, respectively, indicating that a protein and ligand interacted through hydrogen bonds. The principal component analysis (PCA)-based free energy landscape (FEL) of the simulation trajectories also validated a stable combination of four pairs (Supplementary material Fig. **S1**). The MMPBSA approach breaks down the overall binding energy into four separate components: electrostatic interaction, van der Waals interaction, polar solvation, and non-polar solvation interaction. The non-polar solvation term is typically referred to as SASA. Table **6** displays the protein and ligand binding energies. BCL2-B, BCL2-DZY6, IL-1B-DZY6, IL-6-B, PTGS2-DZY6, TNF-B, and TNF-DZY6 all had negative binding free energies of -48.396, -225.275, and -48.396, respectively, in the protein-ligand complex system. Moreover, values of -37.887, -159.809, -260.041, -175.021, and -232.578 KJ/mol indicated the stability of a protein and ligand. The main interaction can possibly be the van der Waals interaction.

## 4 DISCUSSION

Numerous studies have repoted a strong relationship between thyroid function and ovarian function, as well as reproductive physiology. Thyroid hormones exert an impact on follicular development, the metabolism of estrogen and androgen, and the regulation of the menstrual cycle [44]. Preterm birth, miscarriage, and intrauterine fetal death are all at higher risk when hypothyroidism occurs during pregnancy [45], as well as detrimental effects on the offspring’s IQ levels, learning abilities, and neuropsychological functioning [46]. Consequently, thyroid evaluation has become a standard practice for women experiencing infertility, and it is strongly recommended to detect and treat any abnormalities promptly. Numerous studies have consistently demonstrated that appropriate management of subclinical hypothyroidism or hypothyroidism during pregnancy can significantly reduce the risk of obstetric problems, such as miscarriage and preterm birth, ultimately enhancing pregnancy outcomes [47-49].

The preferred treatment for hypothyroidism is levothyroxine [2]. However, it is crucial to avoid the issues of undertreatment and overtreatment [50]. Numerous clinical investigations have shown that TCM is notably effective in treating hypothyroidism and has improved safety profiles and fewer side effects [51, 52]. TCM attributes hypothyroidism to the categories of “consumptive disease” and “goiter”. The occurrence of goiter disease is mostly caused by qi stagnation, phlegm coagulation, blood stasis, and yang deficiency. QUF6 is composed of seven herbs, which jointly play the role of regulating the liver, strengthening the spleen, and tonifying the kidney.

Experiments on humans and animals have repeatedly shown the protective benefits of quercetin on thyroid function [53, 54]. Simultaneously, quercetin and beta-sitosterol exhibit potential as therapeutic agents for managing hypothyroidism in pregnant women [55]. The present study suggests that kaempferol possesses dual properties as a xenobiotic agent, promoting energy expenditure, and as a pharmaceutical compound capable of modulating thyroid hormone activation. Consequently, it has the potential to rectify endocrine and metabolic imbalances in human subjects [56]. It is now known that elevated levels of oxidative stress are linked to hypothyroidism [57]. When compared to normal controls, individuals with subclinical hypothyroidism had considerably higher levels of 7-ketocholesterol, a key byproduct of cholesterol auto-oxidation. Following restoration of therapeutic thyroid function, 7-ketocholesterol levels decreased significantly and were comparable to those observed in the control population [58]. As a key constituent of *Radix rehmanniae* (Shengdihuang), rehmapicrogenin has been extensively studied for its estrogenic properties, thereby providing substantial theoretical evidence supporting its role in maternal pregnancy maintenance [59].

The analysis revealed a total of twenty-five shared targets among QUF6, hypothyroidism, and IVF-ET. Notably, the PPI network analysis demonstrated strong associations among these identified targets. Among them, TNF, IL6, IL1B, BCL2, PTSG2, TP53, and EGF emerged as the top seven potential targets. It is worth mentioning that immunological dysregulation and an increased inflammatory reaction might compromise the integrity of thyroid follicular cells, which can ultimately result in hypothyroidism. Serum levels of TNF-α and interleukin-2 (IL-2) were found to be considerably greater in pregnant women with hypothyroidism than in normal healthy women. Furthermore, it is postulated that alterations in intestinal flora, such as *Prevotella* and *Bacteroides*, may impact the balance of T lymphocytes type 1 (Th1)/T lymphocytes type 2 (Th2) and associated cytokines like TNF-α, potentially leading to thyroid damage [60]. The cytokines IL-1B and IL-6 are essential for various steps of the inflammatory response, leading to the activation and infiltration of neutrophils, monocytes, macrophages, and lymphocytes. A correlation between elevated levels of inflammatory markers and thyroid dysfunction has been documented [61]. In individuals with hypothyroidism, papanas found a favorable correlation between IL-6 levels and thyroxine replacement dosage [62]. However, initial studies have identified biphasic, non-cytotoxic, and reversible effects of IL-1, thereby supporting the involvement of IL-1 in the physiological regulation of thyrocyte function. Elevated concentrations of IL-1 can exert inhibitory effects on thyrocyte function [63]. The genes involved in apoptosis can be categorized into two groups: pro-apoptotic genes (*e.g*., TP53, *etc*.) and anti-apoptotic genes (*e.g*., BCL2, *etc*.) [64]. A study determining how nickel causes thyroid cell death in mice discovered that the expression of Bax protein was up-regulated and the BCL2 was down-regulated. These findings suggest that nickel sulfate (NiSO4) may modulate the mRNA levels of Bax, BCL2, fatty acid synthase (Fas), and caspase-3 in rats, potentially contributing to one of the pathways involved in thyroid tissue apoptosis [65]. Thyroid hormone deficiency can have a major effect on how the central nervous system develops, including cognitive processes like memory and learning [66]. The TP53 protein is essential for basic biological functions like cell cycle control and apoptosis [67], and it is intricately associated with neuronal cell death [68]. The findings of the study demonstrated that overexpression of sirtuin 1 (SIRT1) leads to inhibition of TP53 expression, thereby reducing apoptosis induced by congenital hypothyroidism and improving rat behavior [69]. The continuous stimulation of thyrocyte proliferation by EGF was observed, leading to the dedifferentiation of adult thyrocytes and highlighting its crucial regulatory role in thyrocytes [70]. Studies have found that TP53 is associated with repeated implant failure [71]. Gene polymorphisms in the TP 53 pathway can also impact pregnancy outcomes in IVF [72, 73]. EGFR-dependent autophagy mechanism and the heparin-binding EGF-like growth factor (HB-EGF) are crucial for the loss of neurons and functional impairment in the cerebellum during developing hypothyroidism [74].

According to KEGG analysis, the pathways primarily involved the AGE-RAGE signaling pathway, fluid shear stress and atherosclerosis, lipid and atherosclerosis, TNF signaling pathway, HIF-1 signaling pathway, MAPK signaling pathway, and IL-17 signaling pathway. Thyroid hormone disorders modify the contractility of cardiomyocytes, ultimately leading to heart failure. Hypothyroidism can induce both systolic and diastolic dysfunction in the cardiac muscle, elevate peripheral vascular resistance, and impair endothelial function. There is compelling evidence that hypothyroidism raises the risk for coronary artery disease (CAD) [75, 76]. Increased phosphorylation of the extracellular signal-regulated kinases 1 and 2 (ERK1/2) and MAPK pathways is caused by congenital hypothyroidism, which results in long-lasting changes in hippocampus synaptic function and, eventually, learning and memory impairments [77]. According to molecular docking studies, each core target showed good binding activity with its associated active component, thereby corroborating the reliability of the network pharmacology analysis outcomes. Meanwhile, based on the RMSD, SASA, and Rg analysis in MD stimulations, we validated that the six pairs with the highest docking scores in molecular docking analysis possessed adequate dynamic stability and flexibility. The interactions of proteins and ligands were found to be mainly based on hydrogen bonds and van der Waals interaction.

However, this study has some limitations. Based on the dynamic cross-correlation map (DCCM) and probability density function (PDF), as well as the analysis of conformational change and simulation interaction analysis of the simulated complexes, this study exclusively investigated the pharmacological mechanism of QUF6 for treating hypothyroidism in IVF-ET patients. Subsequently, the druggability assessment should be conducted through animal and cellular experiments in the future [78]. Finally, clinical trials should be carried out to evaluate the safety and clinical efficacy of QUF6.

## CONCLUSION

Exploring the treatment of QUF6 for hypothyroidism caused by IVF-ET has profound implications. Our study investigated the therapeutic benefits of QUF6 in populations undergoing IVF-ET who were experiencing hypothyroidism using a network pharmacology method. Quercetin, beta-sitosterol, kaempferol, 7-ketocholesterol, and rehmapicrogenin were found to be the main active ingredients in QUF6. TNF, IL6, IL1B, BCL2, PTSG2, TP53, and EGF may be the important targets of QUF 6 in treating hypothyroidism patients with IVF-ET. The activation of the AGE-RAGE, TNF, HIF-1, MAPK, and IL-17 signaling pathways was found to be related to its potential processes, and molecular docking and molecular dynamics simulations were used to validate our results. These conclusions lack experimental confirmation and are based on data from databases that are already in existence. Therefore, more experimental investigations, either *in vitro* or *in vivo*, are required to confirm the veracity of the current research findings.

## Figures and Tables

**Fig. (4):**
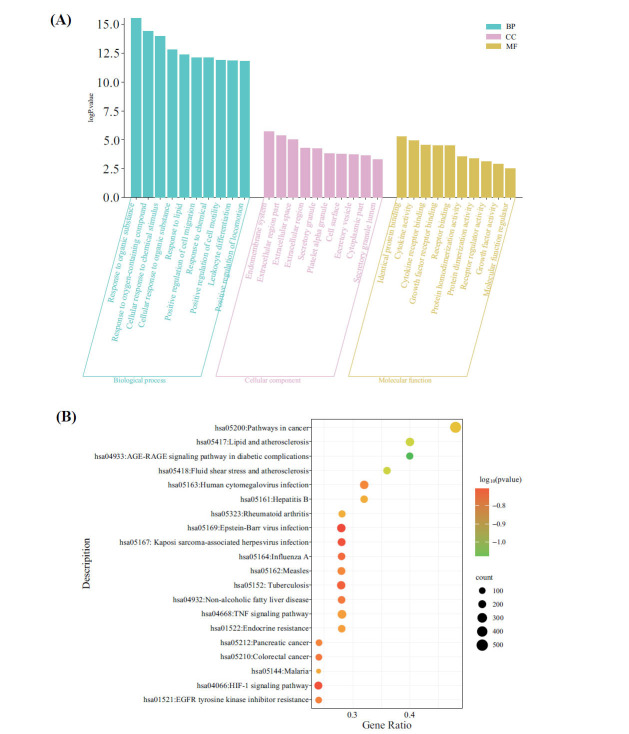
Gene ontology and Kyoto Encyclopedia of Genes and Genomes pathway enrichment analysis. (**A**) Gene ontology analysis; (**B**) Kyoto Encyclopedia of Genes and Genomes pathway enrichment analysis.

**Fig. (6):**
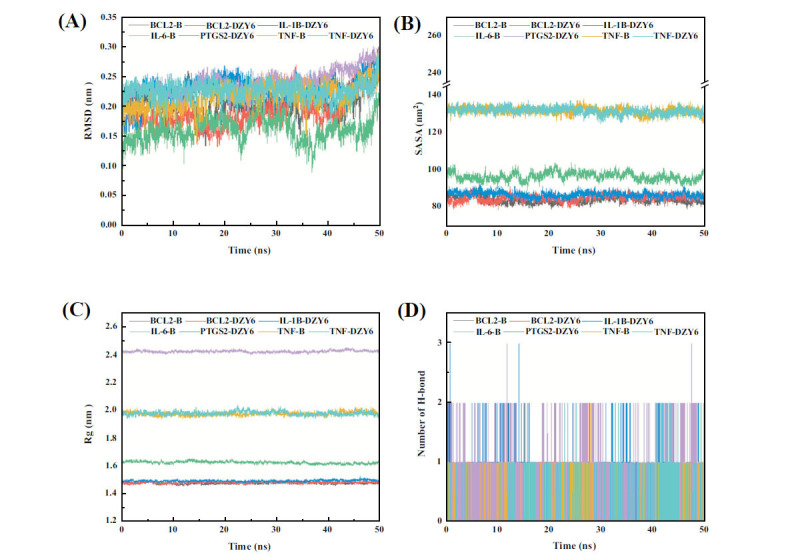
Molecular dynamics (MD) simulations of the main targets and active components of QUF6. (**A**) Changes in the root-mean-square deviation (RMSD) of protein over time; (**B**) Changes in the solvent-accessible surface area (SASA) of protein over time; (**C**) Changes in the radius of gyration (Rg) of protein over time; (**D**) Changes in the hydrogen bonds of protein over time.

**Table 1:** Active ingredients of QUF6.

**Herb**	**Active Ingredients**
Gardeniae Fructus (Zhizi)	Crocetin, (4aS,6aR,6aS,6bR,8aR,10R,12aR,14bS)-10-hydroxy-2,2,6a,6b,9,9,12a-heptamethyl-1,3,4,5,6,6a,7,8,8a,10,11,12,13,14b-tetradecahydropicene-4a-carboxylic acid, Ammidin,Sudan III, Quercetin, Beta-sitosterol, Kaempferol, Stigmasterol,Mandenol, Supraene, Isoimperatorin, Ethyl oleate (NF), 5-hydroxy-7-methoxy-2-(3,4,5-trimethoxyphenyl)chromone, 3-Methylkempferol, GBGB
*Artemisiae scopariae herba* (Yinchen)	Isorhamnetin, Beta-sitosterol, Areapillin, Genkwanin, Skrofulein, Isoarcapillin, Eupalitin, Eupatolitin, Capillarisin, 4'-methylcapillarisin, Demethoxycapillarisin, Artepillin A, Quercetin
*Rehmanniae radix praeparata* (Shudihuang)	Sitosterol, Stigmasterol
*Atractylodes macrocephala* Koidz (Baizhu)	12-senecioyl-2E,8E,10E-atractylentriol, 14-acetyl-12-senecioyl-2E,8E,10E-atractylentriol, 14-acetyl-12-senecioyl-2E,8Z,10E-atractylentriol, α-Amyrin, (3S,8S,9S,10R,13R,14S,17R)-10,13-dimethyl-17-[(2R,5S)-5-propan-2-yloctan-2-yl]-2,3,4,7,8,9,11,12,14,15,16,17-dodecahydro-1H-cyclopenta[a]phenanthren-3-ol, 3β-acetoxyatractylone, 8β-ethoxy atractylenolide
Mori Follum (Sangye)	Poriferast-5-en-3beta-ol, Scopolin, Beta-carotene, Albanol, Inophyllum E, 26-hydroxy-dammara-20,24-dien-3-one, Isoramanone, Moracin B, Moracin C, Moracin D, Moracin E, Moracin F, Moracin G, Moracin H, 4-prenylresveratrol, FA, Oxysanguinarine, Quercetin, Beta-sitosterol, Kaempferol, Stigmasterol, Arachidonic acid, Supraene, Iristectorigenin A icosa-11,14,17-trienoic acid methyl ester, Norartocarpetin, Linolenic acid ethyl ester, Tetramethoxyluteolin, Skimmin (8CI)
*Lophatherum gracile* (Danzhuye)	(4R,4aS,6aS,6aS,6bR,8aR,12aS,14aS,14bR)-4,4a,6a,6b,8a,11,11,14a-Octamethyl-2,4,5,6,6a,7,8,9,10,12,12a,13,14,14b-tetradecahydro-1H-picen-3-One, Cymarin, (3S,5S,8S,9S,10S,13R,14S,17R)-5,14-dihydroxy-3-[(4S,5R,6R)-5-Hydroxy-4-methoxy-6-Methyloxan-2-Yl]Oxy-13-methyl-17-(5-oxo-2H-furan-3-Yl)-2,3,4,6,7,8,9,11,12,15,16,17-dodecahydro-1H-cyclopenta[A]phenanthrene-10-carbaldehyde, Spectrum_000719, Friedelin, 7-ketocholesterol
*Radix rehmanniae* (Shengdihuang)	(5R)-5-methoxypyrrolidin-2-One,1-((2r,5r)-3,4-dihydroxy-5-hydroxymethyl-tetrahydro-furan-2-yl)-1h-pyrimidine-2,4-dione, Salidroside, 7-Hydroxyisoquinoline, Coniferin, Guanosine, Uridine, Isoacteoside, 5-hydroxy-2-methylpyridine, Adenine, Beta-sitosterol, 2-Amino-9-[(2R,3R,4S,5R)-3,4-dihydroxy-5-(Hydroxymethyl)Oxolan-2-Yl]-3H-purin-6-One, (24R)-5-Ergosten-3, (3S,8S,9S,14S,17R)-17-[(2R,5R)-5,6-dimethylheptan-2-Yl]-10,13-dimethyl-2,3,4,7,8,9,11,12,14,15,16,17-dodecahydro-1H-cyclopenta[A]phenanthren-3-Ol, 5959-35-3, Rehmaglutin A, Schembl170902, (Rel)-(2S,5S)-9-[4,4-bis-(hydroxymethyl)-5-methyl-tetrahydro-Furan-2-Yl]Adenine, Indole-3-carboxylic Acid, 135447-39-1, Schembl17580247, (2R)-2-[[(1R,2S,6S)-5-hydroxy-2-(hydroxymethyl)-3,9-dioxatricyclo[4.4.0.02,4]Dec-7-En-10-Yl]Oxy]-6-(Hydroxymethyl)Oxane-3,4,5-Triol (2R,3S,4S,5R,6S)-2-(Hydroxymethyl)-6-[4-[(Z)-3-Hydroxy(2,3-13C2)Prop-1-Enyl]-2-Methoxyphenoxy]Oxane-3,4,5-Triol, Adenosine, 5-Methoxypyrrolidin-2-One, Rehmapicrogenin, Acteoside

**Table 2:** TOP 5 active constituents of QUF6.

**-**	**Ingredients**	**Degree**	**Drug**
A	Quercetin	15	ZZ YC SY
SDH14	Rehmapicrogenin	7	SDH
C	Kaempferol	6	ZZ SDH SY
DZY6	7-Ketocholesterol	6	DZY
B	Beta-sitosterol	5	ZZ YC SY SDH

**Note:** ZZ: Gardeniae Fructus (Zhizi); YC: *Artemisiae scopariae herba* (Yinchen); SY: Mori Follum (Sangye); SDH: *Radix rehmanniae* (Shengdihuang); DZY: *Lophatherum gracile* (Danzhuye).

**Table 3:** The information of nodes in “Herb-constituent-target” (H-C-T) network.

**Node**	**MOL ID**	**Molecular Name**	**OB**	**DL**	**Origin**
ZZ1	MOL004561	SudanIII	84.07	0.59	Gardeniae Fructus (Zhizi)
ZZ2	MOL003095	5-hydroxy-7-methoxy-2-(3,4,5-trimethoxyphenyl)Chromone	51.96	0.41	Gardeniae Fructus (Zhizi)
YC1	MOL000354	Isorhamnetin	49.6	0.31	*Artemisiae scopariae herba* (Yinchen)
YC2	MOL004609	Areapillin	48.96	0.41	*Artemisiae scopariae herba* (Yinchen)
YC3	MOL005573	Genkwanin	37.13	0.24	*Artemisiae scopariae herba* (Yinchen)
YC4	MOL008039	Isoarcapillin	57.4	0.41	*Artemisiae scopariae herba* (Yinchen)
YC5	MOL008040	Eupalitin	46.11	0.33	*Artemisiae scopariae herba* (Yinchen)
YC6	MOL008041	Eupatolitin	42.55	0.37	*Artemisiae scopariae herba* (Yinchen)
YC7	MOL008043	Capillarisin	57.56	0.31	*Artemisiae scopariae herba* (Yinchen)
YC8	MOL008046	Demethoxycapillarisin	52.33	0.25	*Artemisiae scopariae herba* (Yinchen)
YC9	MOL008047	ArtepillinA	68.32	0.24	*Artemisiae scopariae herba* (Yinchen)
BZ1	MOL000049	3β-acetoxyatractylone	54.07	0.22	Atractylodes Macrocephala Koidz (Baizhu)
SY1	MOL002773	Beta-carotene	37.18	0.58	Mori Follum (Sangye)
SY2	MOL003847	Inophyllum E	38.81	0.85	Mori Follum (Sangye)
SY3	MOL003856	Moracin B	55.85	0.23	Mori Follum (Sangye)
SY4	MOL003857	MoracinC	82.13	0.29	Mori Follum (Sangye)
SY5	MOL003858	MoracinD	60.93	0.38	Mori Follum (Sangye)
SY6	MOL003859	MoracinE	56.08	0.38	Mori Follum (Sangye)
SY7	MOL003861	MoracinG	75.78	0.42	Mori Follum (Sangye)
SY8	MOL003862	MoracinH	74.35	0.51	Mori Follum (Sangye)
SY9	MOL003879	4-prenylresveratrol	40.54	0.21	Mori Follum (Sangye)
SY10	MOL001439	Arachidonicacid	45.57	0.2	Mori Follum (Sangye)
SY11	MOL003759	IristectorigeninA	63.36	0.34	Mori Follum (Sangye)
SY12	MOL007879	Tetramethoxyluteolin	43.68	0.37	Mori Follum (Sangye)
DZY1	-	(4R,4aS,6aS,6aS,6bR,8aR,12aS,14aS,14bR)-4,4a,6a, 6b,8a,11,11,14a-octamethyl-2,4,5,6,6a,7,8,9,10,12,12a,13,14,14b-Tetradecahydro-1H-picen-3-one	-	-	*Lophatherum gracile* (Danzhuye)
DZY2	-	Cymarin	-	-	*Lophatherum gracile* (Danzhuye)
DZY3	-	(3S,5S,8S,9S,10S,13R,14S,17R)-5,14-dihydroxy-3-[(4S,5R,6R)-5-Hydroxy-4-methoxy-6-methyloxan-2-Yl]Oxy-13-methyl-17-(5-oxo-2H-furan-3-Yl)-2,3,4,6,7,8,9,11,12,15,16,17-dodecahydro-1H-cyclopenta[A]phenanthrene-10-carbaldehyde	-	-	*Lophatherum gracile* (Danzhuye)
DZY4	-	Spectrum_000719	-	-	*Lophatherum gracile* (Danzhuye)
DZY5	-	Friedelin	-	-	*Lophatherum gracile* (Danzhuye)
DZY6	-	7-ketocholesterol	-	-	*Lophatherum gracile* (Danzhuye)
SDH1	-	Salidroside	-	-	*Radix rehmanniae* (Shengdihuang)
SDH2	-	7-hydroxyisoquinoline	-	-	*Radix rehmanniae* (Shengdihuang)
SDH3	-	Coniferin	-	-	*Radix rehmanniae* (Shengdihuang)
SDH4	-	Uridine	-	-	*Radix rehmanniae* (Shengdihuang)
SDH5	-	Isoacteoside	-	-	*Radix rehmanniae* (Shengdihuang)
SDH6	-	5-hydroxy-2-methylpyridine	-	-	*Radix rehmanniae* (Shengdihuang)
SDH7	-	(24R)-5-ergosten-12|A-Ol	-	-	*Radix rehmanniae* (Shengdihuang)
SDH8	-	(3S,8S,9S,14S,17R)-17-[(2R,5R)-5,6-dimethylheptan-2-Yl]-10,13-dimethyl-2,3,4,7,8,9,11,12,14,15,16,17-dodecahydro-1H-cyclopenta[A]phenanthren-14-Ol	-	-	*Radix rehmanniae* (Shengdihuang)
SDH9	-	Rehmaglutin A	-	-	*Radix rehmanniae* (Shengdihuang)
SDH10	-	Indole-3-carboxylic Acid	-	-	*Radix rehmanniae* (Shengdihuang)
SDH11	-	135447-39-1	-	-	*Radix rehmanniae* (Shengdihuang)
SDH12	-	(2R)-2-[[(1R,2S,6S)-5-hydroxy-2-(hydroxymethyl)-3,9-dioxatricyclo[4.4.0.02,4]dec-7-En-10-Yl]oxy]-6-(hydroxymethyl)oxane-3,4,5-Triol	-	-	*Radix rehmanniae* (Shengdihuang)
SDH13	-	(2R,3S,4S,5R,6S)-2-(hydroxymethyl)-6-[4-[(Z)-3-hydroxy(2,3-13C2)prop-1-enyl]-2-methoxyphenoxy]oxane-3,4,5-Triol	-	-	*Radix rehmanniae* (Shengdihuang)
SDH14	-	Rehmapicrogenin	-	-	*Radix rehmanniae* (Shengdihuang)
SDH15	-	Acteoside	-	-	*Radix rehmanniae* (Shengdihuang)
A	MOL000098	Quercetin	46.43	0.28	Gardeniae Fructus (Zhizi) *Artemisiae scopariae herba* (Yinchen) Mori Follum (Sangye)
B	MOL000358	Beta-sitosterol	36.91	0.75	Gardeniae Fructus (Zhizi) *Artemisiae scopariae herba* (Yinchen) Mori Follum (Sangye) *Radix rehmanniae* (Shengdihuang)
C	MOL000422	Kaempferol	41.88	0.24	Gardeniae Fructus (Zhizi) Mori Follum (Sangye)

**Note:** OB: oral bioavailability; DL: drug-like; Since *Lophatherum gracile* (Danzhuye) and *Radix rehmanniae* (Shengdihuang) were not retrieved in TCMSP, its active ingredients were obtained through the BATMAN-TCM. MOLID, OB and DL were unavailable.

**Table 4:** Molecule docking energy of core ingredients of QUF6 and key targets.

**-**	**Resolution**	**A**	**B**	**C**	**DZY6**	**SDH14**
TNF (PDB ID: 5M2J)	2.70A	-7.9	-8.16	-7.51	-9.7	-5.49
IL6 (PDB ID: 1ALU)	2.50A	-6.61	-8.4	-5.73	-7.96	-5.03
IL1B (PDB ID: 31BI)	1.89A	-7.06	-7.88	-7.23	-8.42	-5.07
BCL2 (PDB ID: 4LXD)	1.90A	-5.78	-9.25	-5.87	-9.15	-5.55
PTGS2(PDB ID: 5F19)	2.04A	-7.4	-7.62	-7.64	-9.87	-6.29
TP53 (PDB ID: 8A92)	1.37A	-6.86	-7.55	-7.3	-7.98	-5.01
EGF (PDB ID: 1NQL)	2.80A	-5.53	-7.08	-5.65	-7.08	-4.19

**Note:** A: quercetin; B: beta-sitosterol; C: kaempferol; DZY6: 7-Ketocholesterol; SDH14: rehmapicrogenin.

**Table 5:** Hydrogen bond position information for the QUF6 molecule docking.

**Ligand**	**Molecular Docking Pairs**	**Position**	**Number of Hydrogen Bonds**	**Length**
A: quercetin	TNF-A	ARG-98	2	1.9
				2.4
		LEU-4	2	1.9
				2.2
	IL6-A	ASP-140	1	1.9
		ASN-144	2	2.0
				2.9
	IL1B-A	LEU-82	2	2.2
				2.4
		LEU-80	1	2.0
		1EU-26	3	2.1
				2.1
				2.1
	BCL2-A	ASP-8	2	2.1
				2.4
		HIS-91	1	2.1
		GLU-88	1	2.2
		ASP-193	1	2.1
		TRP-192	1	2.5
	PTGS2-A	GLU-42	1	2.2
		ASN-43	1	2.0
		ASP-125	2	2.0
				2.0
		ARG-469	2	2.0
				2.6
		GLU465	1	2.1
	TP53-A	LEU-29	1	2.2
		LYS-272	1	2.5
		ASP-251	2	2.0
				2.8
		SER-248	2	2.3
				3.5
		SER-246	1	1.9
		ASN-271	3	1.9
				2.0
				2.1
	EGF-A	GLU-578	1	2.0
		THR-601	1	2.1
		TRP-584	2	1.9
				2.6
B: beta-sitosterol	TNF-B	ARG-98	1	2.2
	IL6-B	ARG-179	1	1.9
		ARG-182	2	2.0
				2.6
	IL1B-B	GLN-38	1	2.4
	BCL2-B	GLU-176	1	1.8
	PTGS2-B	LYS-532	1	2.1
		GLN-372	1	1.9
	TP53-B	GLY-245	1	2.1
	EGF-B	GLN-384	1	2.2
C: kaempferol	TNF-C	LEU-4	1	2.0
		VAL-2	2	2.1
				2.5
	IL6-C	PRO-139	1	1.8
		ASN-144	1	2.1
		GLU-99	1	1.7
		GLU-95	1	1.9
		LYS-120	2	1.8
				2.8
	IL1B-C	VAL-132	1	2.7
		LEU-26	2	1.9
				2.3
		LEU-80	1	2.0
		LEU-82	2	2.3
				2.5
	BCL2-C	ASP-8	1	2.3
		HIS-91	2	2.2
				2.3
		ASP-293	1	2.0
	PTGS2-C	GLU465	1	2.1
		GLY-45	1	2.0
		GLN-461	1	2.3
		PRO-154	1	1.8
		CYS-47	2	1.6
				2.2
	TP53-C	ASN-271	1	2.0
		ASN-272	1	2.5
		HIS-269	1	2.1
		SER-256	1	2.0
		SER-248	1	2.4
		ASP-251	2	2.2
				2.7
C: kaempferol	-	PHE-296	1	2.4
		LEU-294	1	2.0
	EGF-C	HIS-597	1	2.1
		THR-601	1	2.1
		THR-581	1	3.4
		GLU-578	2	2.3
				2.5
		ASN-579	1	3.5
DZY6: 7-ketocholesterol	TNF-DZY6	VAL-2	1	3.5
	IL6-DZY6	LEU-62	1	2.5
		ARG-168	1	2.5
	IL1B-DZY6	GLU-64	1	2.1
	BCL2-DZY6	PHE-109	1	2.5
	PTGS2-DZY6	ARG-44	2	2.4
				2.6
	TP53-DZY6	ARG-24	1	2.0
		ASP-88	1	2.3
		ARG-91	2	2.4
				3.5
		GLN-355	1	3.1
		ILE-89	1	3.6
	EGF-DZY6	GLY-574	1	2.7
		MET-244	1	3.5
SDH14: rehmapicrogenin	TNF-SDH14	LEU-26	1	1.9
		GLU-135	1	2.1
		ILE-136	2	1.9
				3.3
	IL6-SDH14	GLU-172	1	2.1
		LEU-62	1	3.1
		LEU-64	2	1.9
				2.1
	IL1B-SDH14	GLN-39	1	1.9
		VAL-41	2	1.9
				2.3
	BCL2-SDH14	ASN-9	1	2.0
		ASN-179	1	2.1
		TYR-7	1	1.8
		GLY-6	1	3.0
		ARG-4	2	2.0
				3.0
		THR-5	1	1.9
SDH14: rehmapicrogenin	PTGS2-SDH14	TRP-387	1	2.5
		TYR-385	1	2.2
		HIS-207	1	2.1
	TP53-SDH14	GLU-71	1	1.9
		ARG-67	1	2.3
	EGF-SDH14	THR-601	1	2.5

**Table 6:** MMPBSA analysis of protein and ligand.

**Energy**	**BCL2-B**	**BCL2-DZY6**	**IL-1B-DZY6**	**IL-6-B**	**PTGS2-DZY6**	**TNF-B**	**TNF-DZY6**
Van der Waals Energy (KJ/mol)	-167.645	-178.914	-141.463	-146.241	-212.314	-136.710	-158.056
Electrostatic energy (kJ/mol)	-101.092	-113.132	-36.829	-275.114	-209.732	-179.653	-242.409
Polar solvation energy (KJ/mol)	180.452	56.794	104.036	214.605	175.183	126.406	161.934
Nonpolar solvation Energy (KJ/mol)	-19.466	-18.720	-17.028	-17.277	-22.802	-17.349	-18.738
Total Binding Energy (KJ/mol)	-107.750	-253.973	-91.285	-224.027	-269.665	-207.305	-257.269
-T∆S (KJ/mol)	59.354	28.698	53.398	64.218	9.625	32.284	24.691
Total Binding Free Energy (KJ/mol)	-48.396	-225.275	-37.887	-159.809	-260.041	-175.021	-232.578

## Data Availability

The authors confirm that the data supporting the findings of this research are available within the article.

## LIST OF ABBREVIATIONS

CAD: Coronary Artery Disease
CC: Cell Components
FEL: Free Energy Landscape
GO: Gene Ontology
HIF-1: Hypoxia-inducible Factor 1
HPO: Hypothalamic-pituitary-thyroid
KEGG: Kyoto Encyclopedia of Genes and Genomes
LH: Luteinizing Hormone
MD: Molecular Dynamics
MF: Molecular Functions
OB: Oral Bioavailability
PPI: Protein-protein Interaction
SASA: Solvent-accessible Surface Area
TCM: Traditional Chinese Medicine
TH: Thyroid Hormone
TNF: Tumor Necrosis Factor
TSH: Thyroid-stimulating Hormone
VMD: Visual Molecular Dynamics
