# Supplementary material for: Mechanisms of a Patented Chinese Herbal Medicine for Treating Hypothyroidism in In Vitro Fertilization-Embryo Transfer: A Combination of Network Pharmacology, Molecular Docking, and Molecular Dynamics Simulation
Source: Curr Pharm Des. 2025 Apr 15;31(35):2849–65. doi: 10.2174/0113816128364578250212094405 (PMC12606615; doi:10.2174/0113816128364578250212094405)
Supplement: Supplementary file 1 [file CPD-31-35-2849_SD1.pdf]

Supplementary Material

Mechanisms of a Patented Chinese Herbal Medicine for Treating Hypothyroidism in *In Vitro* Fertilization-Embryo Transfer: A Combination of Network Pharmacology, Molecular Docking, and Molecular Dynamics Simulation

Chang Liu[1,#], Weihuan Hu[2,#], Tianyi Zhou[2,#], Jue Zhou[3], Fangfang Wang[4], Xiaoling Feng[5,*] and Fan Qu[2,*]

[1]First Clinical Medical College, Heilongjiang University of Chinese Medicine, Harbin, 150040, China; [2]Department of Traditional Chinese Medicine, Women's Hospital School of Medicine Zhejiang University, Hangzhou, 310006, China; [3]College of Food Science and Biotechnology, Zhejiang Gongshang University, Hangzhou, 310018, China; [4]Department of Obstetrics, Women's Hospital School of Medicine Zhejiang University, Hangzhou, 310006, China; [5]Second Department of Gynecology, First Affiliated Hospital of Heilongjiang University of Chinese Medicine, Harbin, 150040, China

1873-4286/25 © 2025 Bentham Science Publishers

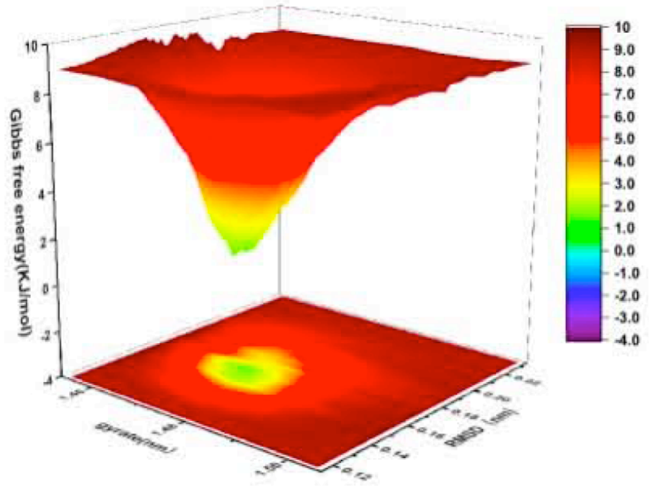

(**A**)

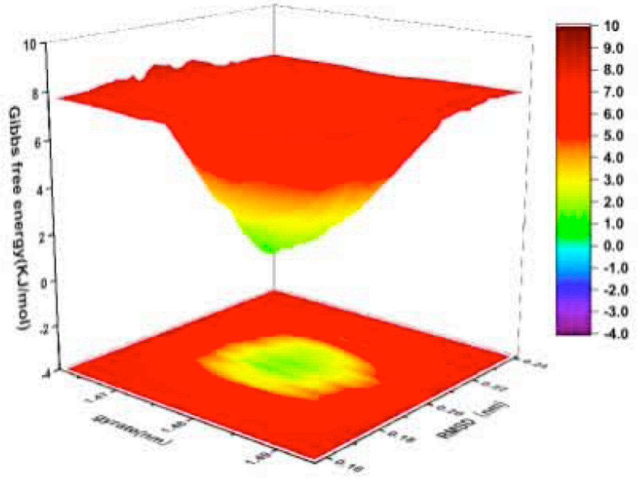

(**B**)

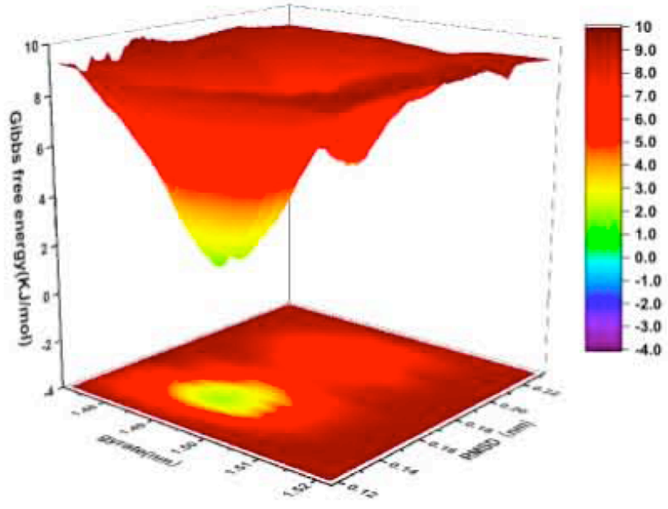

(**C**)

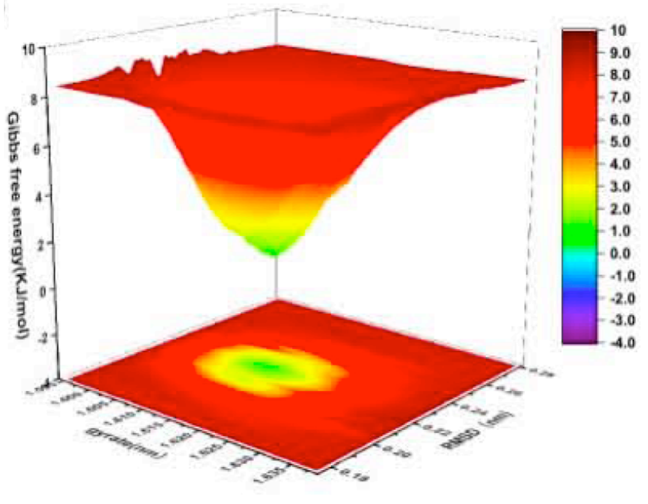

(**D**)

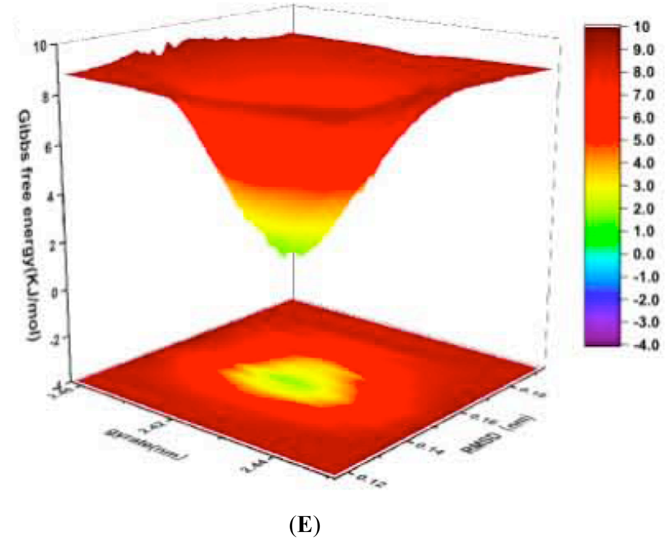

(**E**)

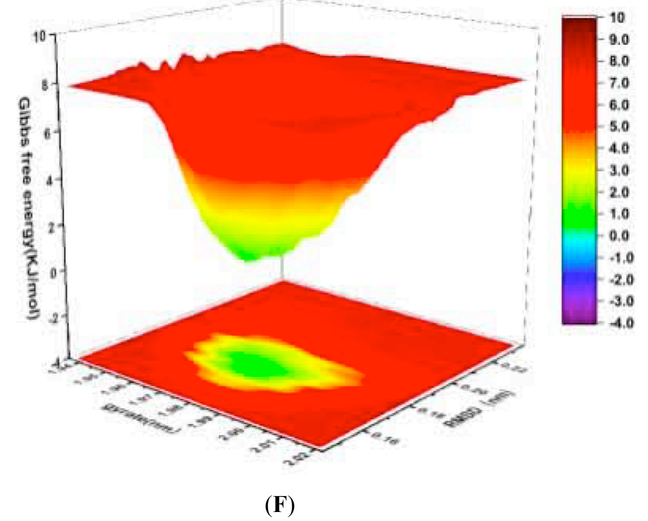

(**F**)

(Fig. S1) Contd….

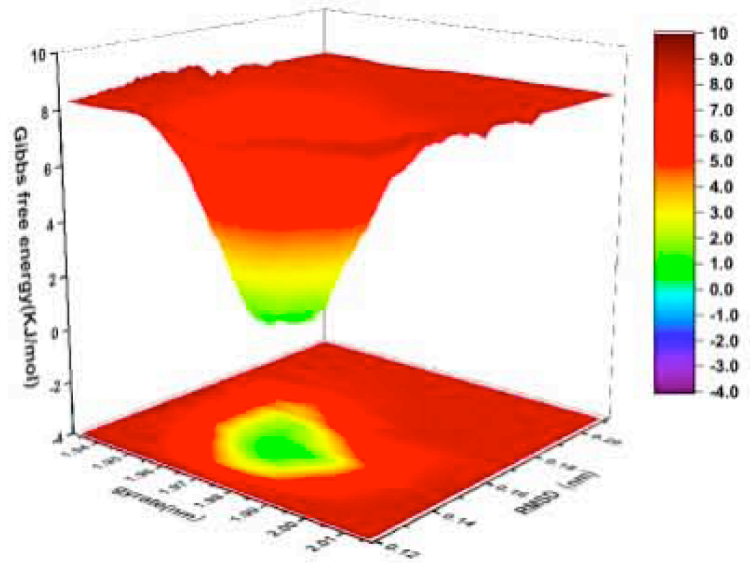

(**G**)

Fig. (S1). The principal component analysis (PCA)-based free energy landscape (FEL) of the simulation trajectories of main targets and active components of QUF6. (**A**) BCL2-B; (**B**) BCL2-DZY6; (**C**) IL-1B-DZY6; (**D**) IL-6-B; (**E**) PTGS2-DZY6; (**F**) TNF-B; (**G**) TNF-DZY6.
